# Screening of novel tumor-associated antigens for lung adenocarcinoma mRNA vaccine development based on pyroptosis phenotype genes

**DOI:** 10.1186/s12885-023-11757-7

**Published:** 2024-01-02

**Authors:** Fang Zhou, Meng Wang, Zheng Wang, Wei Li, Xike Lu

**Affiliations:** Department of Thoracic Surgery, Tianjin Chest Hospital of Tianjin University, 261 Taierzhuang South Road, Jinnan District, Tianjin, 300222 China

**Keywords:** Lung adenocarcinoma, mRNA vaccine, Pyroptosis, Tumor immune microenvironment, Immune landscape

## Abstract

This study aimed to identify new pyroptosis-associated tumor antigens for use in mRNA vaccines and the screening of sensitive LUAD populations suitable for vaccination. The association between tumor immune infiltrating cell abundance and potential tumor antigens was investigated and visualized using the analysis modules of gene expression, clinical outcomes, and somatic copy number variation. In addition, the pyroptosis-related genes (PRGs) were clustered, the relative pyroptosis subtypes (PSs) and gene modules were identified, and the prognostic value of the PSs was examined. The expression of key PRGs in two lung adenocarcinoma cell lines was verified by RT-qPCR. Four tumor pyroptosis-associated antigens, CARD8, NAIP, NLRP1, and NLRP3, were screened as potential candidates for LUAD mRNA vaccine development. In the construction of consensus clusters for PRGs, two PSs, PS1 and PS2, were classified, in which patients with PS1 LUAD had a better prognosis. In contrast, patients with PS2 LUAD may have better responsiveness to mRNA vaccine treatment. The key PRGs can be regarded as biomarkers to predict the LUAD prognosis and identify patients suitable for mRNA vaccines. The RT-qPCR results showed that the expression levels of CSMD3, LRP1B, MUC16 and TTN were significantly increased in the two lung adenocarcinoma cell lines, while the expression levels of CARD8, TP53 and ZFHX4 were significantly reduced. The antigens CARD8, NAIP, NLRP1, and NLRP3, which are associated with tumor pyroptosis, could be candidate molecules for LUAD mRNA vaccine development. Patients with PS2 LUAD may be suitable candidates for mRNA vaccine treatment.

**Supplementary Information:**

The online version contains supplementary material available at 10.1186/s12885-023-11757-7.

## Introduction

Lung cancer (LC) is a common and fatal disease with approximately 2.09 million new cases per year and an increasing incidence and mortality rate [1]. In China, the number of new cases and deaths due to LC ranks first among all types of cancer [2]. Lung adenocarcinoma (LUAD) accounts for 40% of all LCs ADDIN EN.CITE [2, 3]. Although the clinical prognosis of patients with LUAD has improved with advances in diagnosis, surgery, radiotherapy, and molecular therapy ADDIN EN.CITE [4], the 5-year survival rate of LUAD remains low ADDIN EN.CITE [5, 6]. Therefore, there is a need to investigate the molecular mechanisms of LUAD in detail to identify new therapeutic targets for its treatment.

**Fig. 1:**
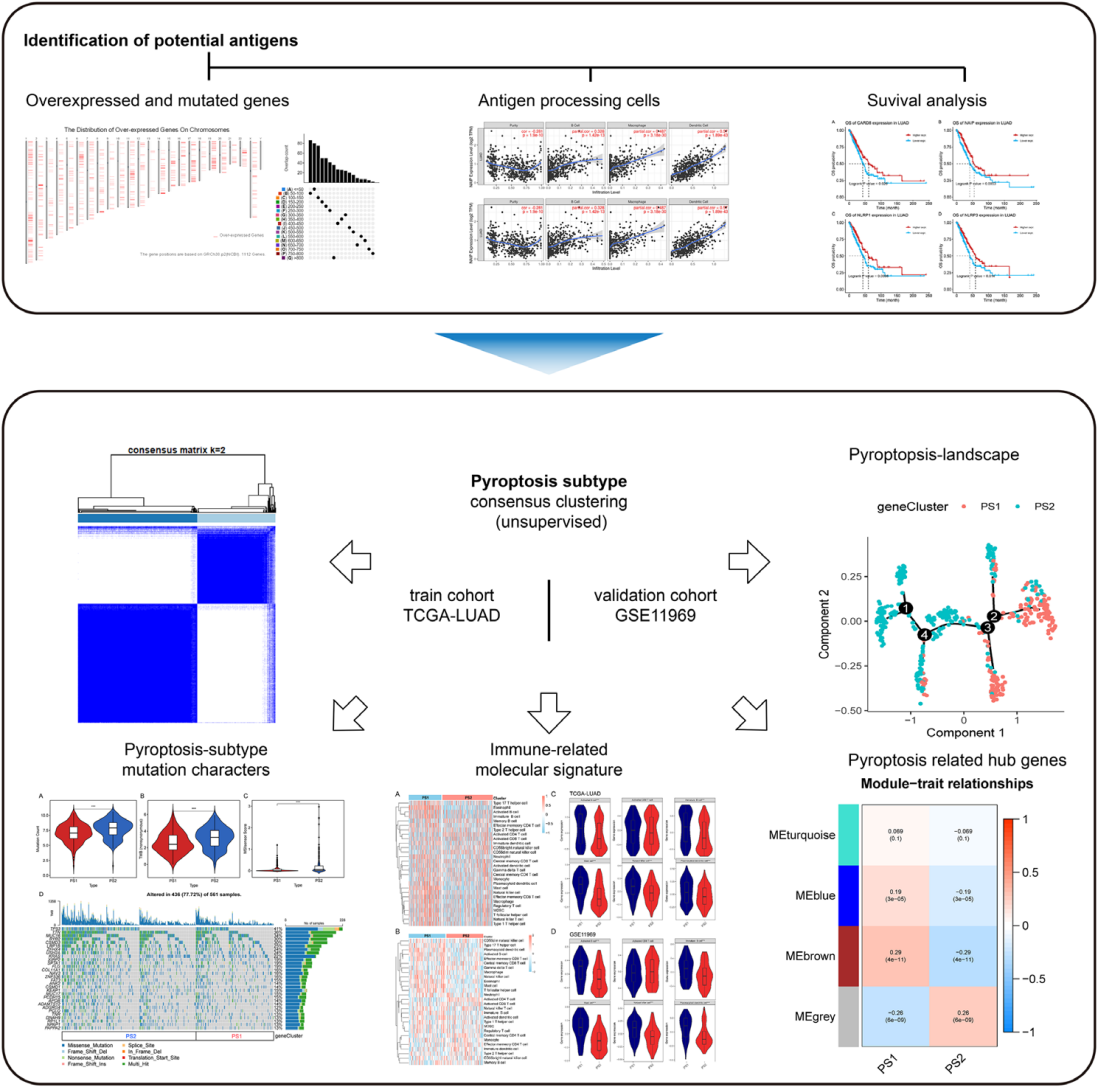
Project analysis process chart

Pyroptosis is a recently discovered mode of programmed cell death prevalent in tumor cells. The primary biochemical feature is the formation of inflammatory vesicles. Pyroptosis plays a dual role in tumor development [7]. On the one hand, it inhibits tumorigenesis and progression. On the other hand, pyroptosis provides suitable conditions for tumor cell growth; it is also closely associated with tumor growth. Therefore, an in-depth study of pyroptosis could provide a new direction for developing novel tumor treatment strategies. Recently, it was found that the proliferation of tumors could be significantly inhibited by inducing pyroptosis in LUAD cells [8].

In recent times, cancer vaccines have received increasing attention from researchers in the treatment of cancer, as they have a better therapeutic effect, a wider therapeutic window, and fewer toxic side effects compared with traditional chemotherapy and immunotherapy [9], which are effective treatment methods for tumors. With the development of second-generation sequencing and bioinformatics technology, it has been possible to discover an increasing number of new tumor antigens. Owing to the heterogeneity of tumors and the diversity of tumor epitopes among individuals, the development of individualized cancer vaccines has become important [10]. Cancer vaccines under research and development can be classified according to antigen types, such as tumor cells, DNA, RNA, dendritic cells, and peptides [11]. Based on the comparative results, mRNA vaccines have several advantages. For example, the synthesis of mRNA vaccines has the advantage of being convenient, inexpensive, rapid, and arbitrarily modified encoded proteins could be sequenced, designed, and obtained by synthesizing and transcribing mRNA sequences in vitro. This allows individualized and precise treatment of tumors compared to traditional peptide vaccines, which require the analysis of cancer genome sequences, incurring additional costs to the treatment regime [12, 13]. In addition, mRNA vaccines can be delivered via multiple delivery methods based on peptides, lipids, or polymers [14], and RNA sequence modifications allow for different mRNA half-lives [15]. Furthermore, mRNA has no risk of gene integration compared to DNA vaccines and can prevent insertion or deletion mutations in genes [12]. In addition, mRNA vaccines can induce humoral and cellular immunity in the body, reducing the possibility of resistance to cancer vaccines [16]. Therefore, mRNA vaccines targeting tumor-associated antigens may potentially have tumor therapy applications. However, few studies on mRNA vaccines have been reported in the field of LUAD therapy. Therefore, the potential population that could benefit from mRNA vaccines is currently unclear, and there is a need to screen for suitable tumor-associated antigens for vaccine development.

This study investigated genes related to pyroptosis in patients with LUAD based on identifying pyroptosis-related tumor antigens for mRNA vaccine production to kill tumor cells by activating tumor pyroptosis. Patients with LUAD were typed according to the pyroptosis-related genes (PRGs) to screen for those sensitive to this type of mRNA vaccine treatment, selecting the subtype of patients suitable for vaccination. In addition, mutations in patients with different subtypes and immune statuses were identified. Finally, using the “WGCNA” package in R, hub genes that could be used as markers to monitor the pyroptosis status of patients and assess their prognosis were screened. Figure 1 shows a flowchart that illustrates the project analysis process.

## Methods and materials

### Data acquisition and processing

The LUAD dataset of The Cancer Genome Atlas (TCGA; n = 585) was obtained from the UCSC Xena database. The data types counting and fragments per kilobase of exon per million mapped fragments were selected; “primary solid tumor” (01 A) were extracted from these and converted into transcript per million formats. The data of the “masked somatic mutation” were selected as the somatic mutation data of LUAD and were preprocessed using the “VarScan” package in R software. The data on age, Tumor Node Metastasis (TNM) stage, survival time, and survival status were downloaded. Patients lacking clinical information were excluded, and 513 patients with survival information and 507 with other clinical information were retained. GSE11969 [17] and the gene level and survival time data were obtained from the GEO database, and the data samples were obtained from *Homo sapiens*. The microarray platform was based on GPL7015. A total of 163 tumor samples were retained, and the microarray data were normalized using the “Limma” package in R software [18].

In addition, PRGs were extracted from GeneCards (https://www.genecards.org/), including all genes with a score > 0.15, resulting in the inclusion of 403 PRGs (Table S1). Furthermore, immunogenic cell death (ICD) genes and immune checkpoint (ICP) genes were obtained from previous studies (Tables S2 and S3) [19, 20].

### Identification of tumor antigens

#### Gene expression profiling interactive analysis and cBioPortal analysis

Raw RNA-seq data from the TCGA database were recalculated based on UCSC Xena to avoid inefficient variance analysis. Gene expression profiling interactive analysis was carried out [21] to integrate differential gene levels. The differentially expressed genes (DEGs) were determined by |log_2_FC| > 1 and q < 0.01. The overall survival (OS) rate was assessed using the K-M method, with the threshold value as the median. Statistical significance was set at *P* < 0.05.

Raw RNA-seq data were integrated to compare genetic alterations in LUAD [22]. In addition, tumor mutation burden (TMB) data were extracted for TCGA-LUAD patients. Statistical significance was set at *P* < 0.05.

### Tumor antigens and immune cell infiltration

The association between tumor immune infiltrating cell abundance and potential tumor antigens was determined by the gene expression results and somatic copy number variation via the tumor immune estimation resource [23]. Purity adjustments were selected based on the Spearman analysis. The statistical threshold was set at *P* > 0.05.

In addition, MCPcounter [24] was used to estimate the infiltration of immune cells in tumors from expression data, which offers information on abundance of eight types of immune cells, including CD4 + T cells, CD8 + T cells, natural killer cells, B cells, and monocytes. The abundance of fibroblasts and endothelial cells was calculated using this method. Spearman correlation analysis was performed based on MCPcounter abundance estimates, and antigen gene expression (*P* < 0.05) was regarded as a significantly different judgment criterion.

### Discovery of the pyroptosis subtype

The “ConsensusClusterPlus” package in R software was used to cluster PRGs with regard to their expression profiles [25]. The package creates clusters according to their expression profiles. The corresponding pyroptosis subtype (PS) was determined based on the consistency matrix. Partitioning was performed using a median algorithm with a “1-Pearson” distance measure, and 200 replications were conducted, with 80% of the patients in the group being resampled each time. The clustering set ranged from two to nine, and optimal partitioning was defined based on the consensus matrix. The PS was verified based on an independent GSE11969 cohort with the same setting.

### Prognostic evaluation of the PS

The log-rank test was used to determine the prognostic value of the PSs. An ANOVA was used to evaluate the correlation between PSs and cellular features associated with pyroptosis. The Chi-squared test was used to screen for frequently mutated genes. Using the “GSVA” package [26], ssGSEA was used to calculate the immune enrichment score of each sample.

### Gene co-expression network

The “WGCNA” package in R software [27] was used to screen for PRGs. The soft threshold was determined based on the pickSoftThreshold module and was found to be six. Based on this threshold, the network and a topology matrix were constructed and the eigengenes were calculated. Intermodule correlations were determined based on module eigengenes, and hierarchical clustering was performed. Functional enrichment analysis was conducted using the Metascape database (www.metascape.org/) [28]. which mainly includes the Kyoto Encyclopedia of Genes and Genomes and Gene Ontology analyses [29, 30]. Statistical significance was set at *P* < 0.05.

### Construction of a pyroptosis landscape in the Tumor microenvironment

Using the “Monocle” package in R software with a Gaussian distribution [31], graph learning was used to visualize the distribution of PSs in patients. The maximum number of components was set to four, and the discriminative reduction method of “DDRtree” was applied. Finally, the pyroptosis landscape was visualized using a color-coded PS functional map of cell trajectories.

### RNA extraction and RT-qPCR

Real-time polymerase chain reaction (RT-qPCR) was used to analyze mRNA expression. Specific primers for human-derived key genes (Table S4) were designed to extract total RNA from lung adenocarcinoma cell lines ((A549 and XWLC-05) (iCell Bioscience Inc, Shanghai, China)) and normal human bronchial epithelial cells ((BEAS-2B) (iCell Bioscience Inc, Shanghai, China)) with TRIzol (Invitrogen, Carlsbad, California, USA). After identifying purity and concentration, RNA is reverse-transcribed to cDNA. The expression of the gene of interest was then quantitatively analyzed by the qPCR fluorescence kit (AceQTM Universal SYBR Qpcr Master Mix, Nanjing, China). GAPDH as an internal reference. Relative gene expression was determined by comparing the 2^−ΔΔCt^ method.

### Statistical analysis

All statistical analyses were performed using R software (version 4.1.1). The W-K test was used to evaluate differences between two groups. The K-W test was used to determine differences between multiple groups. Spearman analysis was used for correlation analysis. The gene expression of cell lines was statistically analyzed using one-way ANOVA from unpaired samples. Statistical significance was set at *P* < 0.05.

## Results

### Identification of potential pyroptosis-associated antigens in LUAD

**Fig. 2:**
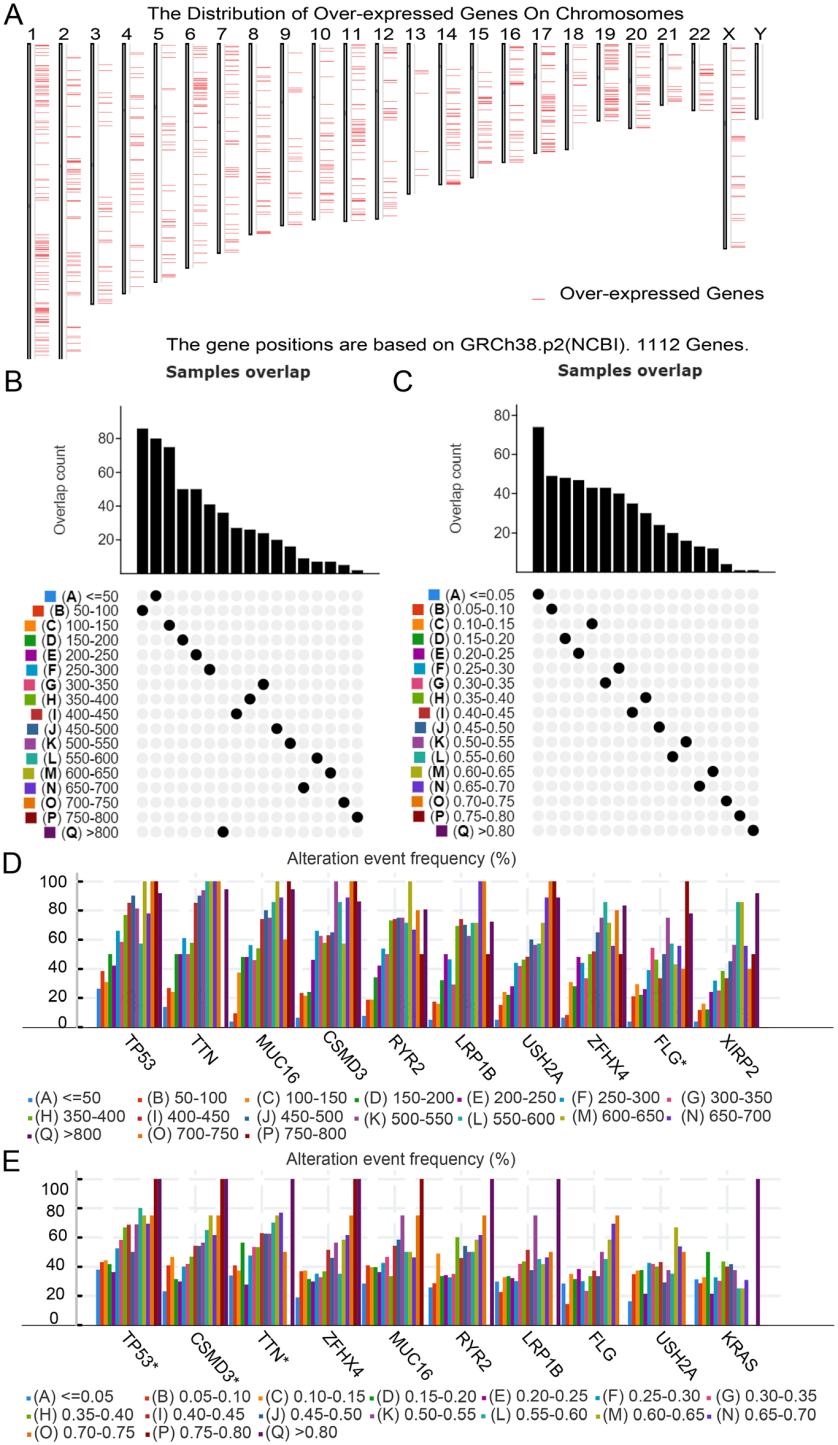
Discrimination of potential tumor antigens in lung adenocarcinoma (LUAD). (**A**) Tumor-related antigens in LUAD. The chromosomal distribution of differentially expressed genes in LUAD is suggested. (**B**)–(**E**) Tumor-specific antigens in LUAD. (**B**, **C**) Samples overlapping in altered genomic segment groups (each column represents a different sample, and the y-axis represents the overlap count of mutated gene fragments in the genome with tumor-specific antigens. This overlap count indicates how many common mutations exist between a specific gene fragment in the genome and genes that potentially encode tumor-specific antigens in each sample). Genes with the highest frequency in altered genomic fragments (**D**) and mutation count groups (**E**) (the X-axis represents the samples, with each sample corresponding to an individual in LUAD patients. Each bar represents a sample, and each color represents the mutation status of the gene in different samples). LUAD, lung adenocarcinoma

First, abnormally expressed genes were identified by TCGA-LUAD analysis, and 4,469 DEGs were detected, of which 1,112 may encode tumor-associated antigens for overexpression (Fig. 2A). Mutated gene segments and mutation counts in LUAD tumor samples were analyzed by VarScan software, and a total of 17,274 mutated genes that may encode tumor-specific antigens were screened based on the determination of mutated gene fragments and mutation counts (Fig. 2B and D). In mutation analysis revealed that TP53 was the most commonly mutated gene in mutant genomic fragments and mutation counts (Fig. 2C and E**)**. In addition, significantly high mutation number and mutation frequency were observed for TP53, TTN, CSMD3, ZFHX4, MUC16, and LRP1B in LUAD. With regard to the full-length TTN and MUC16 genes, their specificity was not high. In contrast, mutations in other genes were thought to be significant in LUAD.

Combining the results of 17,274 mutated and 1,112 overexpressed genes, 800 genes were identified as frequently mutated and upregulated cancer-related genes, which may serve as potential tumor antigens (Supplementary Fig. 1).

**Fig. 3:**
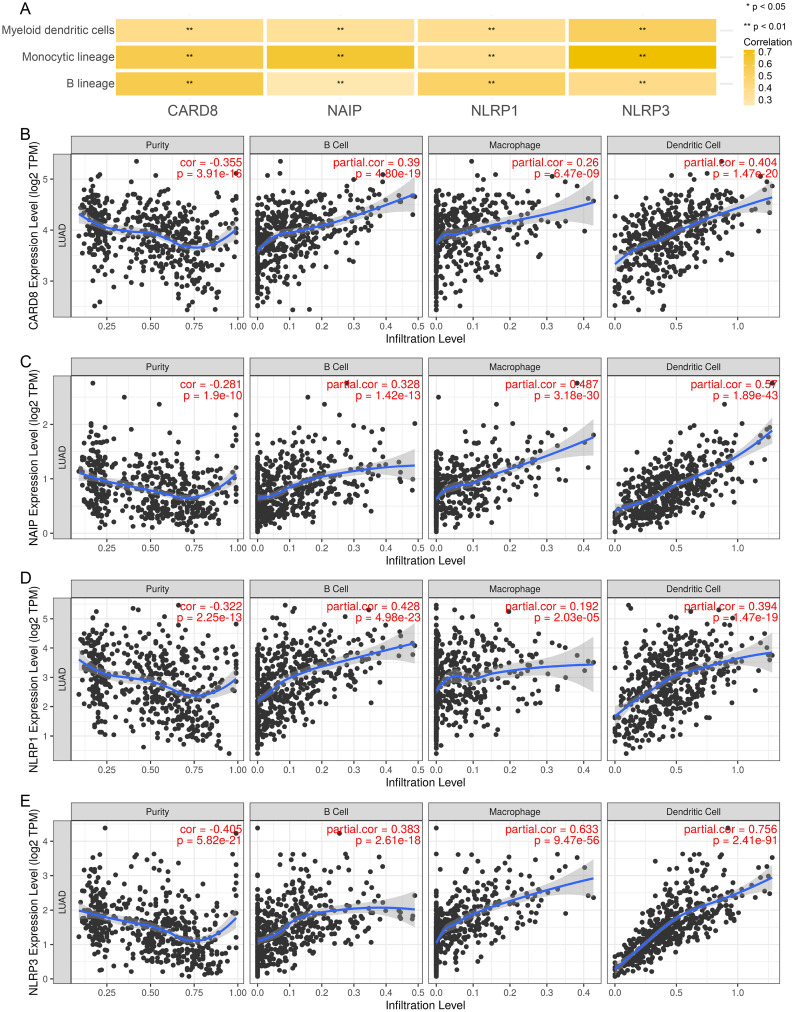
Discrimination of tumor antigens related to APCs. (**A**) Correlation analysis of APCs with CARD8, NAIP, NLRP1, and NLRP3, calculated by MCPcounter. (**B**)–(**E**) Correlation of CARD8 (**B**), NAIP (**C**), NLRP1 (**D**), and NLRP3 (**E**) expression levels with macrophage, dendritic cell, and B-cell infiltration in LUAD tumors. *P < 0.05, **P < 0.01. LUAD, lung adenocarcinoma

**Fig. 4:**
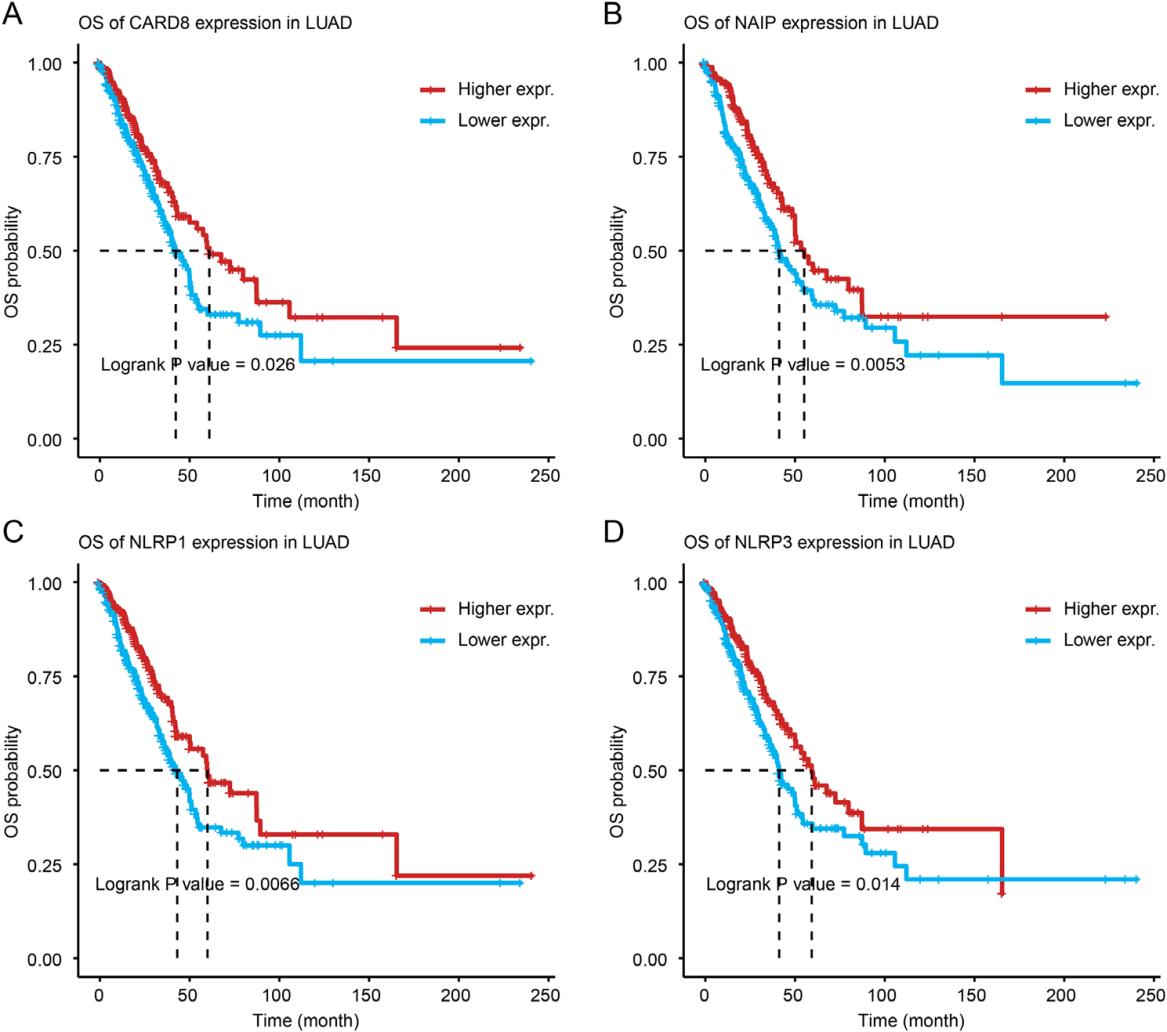
Discrimination of tumor antigens related to lung adenocarcinoma (LUAD) prognosis. (**A**)–(**D**) overall survival of LUAD cases stratified by CARD8 (**A**), NAIP (**B**), NLRP1 (**C**), and NLRP3 (**D**) expression levels. LUAD, lung adenocarcinoma; OS, overall survival

Subsequently, the possibility of using pyroptosis genes as mRNA antigens was explored. We intersected 800 potential tumor antigens with 403 PRGs and obtained 36 mutation-characterized PRGs. Considering the interaction between tumor antigens and antigen-producing cells, the correlation of these antigens with antigen-presenting cells (APCs) was further screened using MCPcounter in combination with the TIMER database. As a result, it was found that the CARD8, NAIP, NLRP1, and NLRP3 genes were significantly positively correlated with various antigen-producing cells (Fig. 3A), which corroborated the results of the TIMER database, where the CARD8, NAIP, NLRP1, and NLRP3 genes were positively correlated with B and dendritic cells (Fig. 3B and E). In addition, a better OS prognosis was found in the group with high-expression of CARD8, NAIP, NLRP1, and NLRP3 (Fig. 4A and D). The expression data showed various trends in different TNM stages and the expression of these four genes tended to decrease in the late stages (Supplementary Fig. 2A–2D).

### Identification of potential PSs of LUAD

**Fig. 5:**
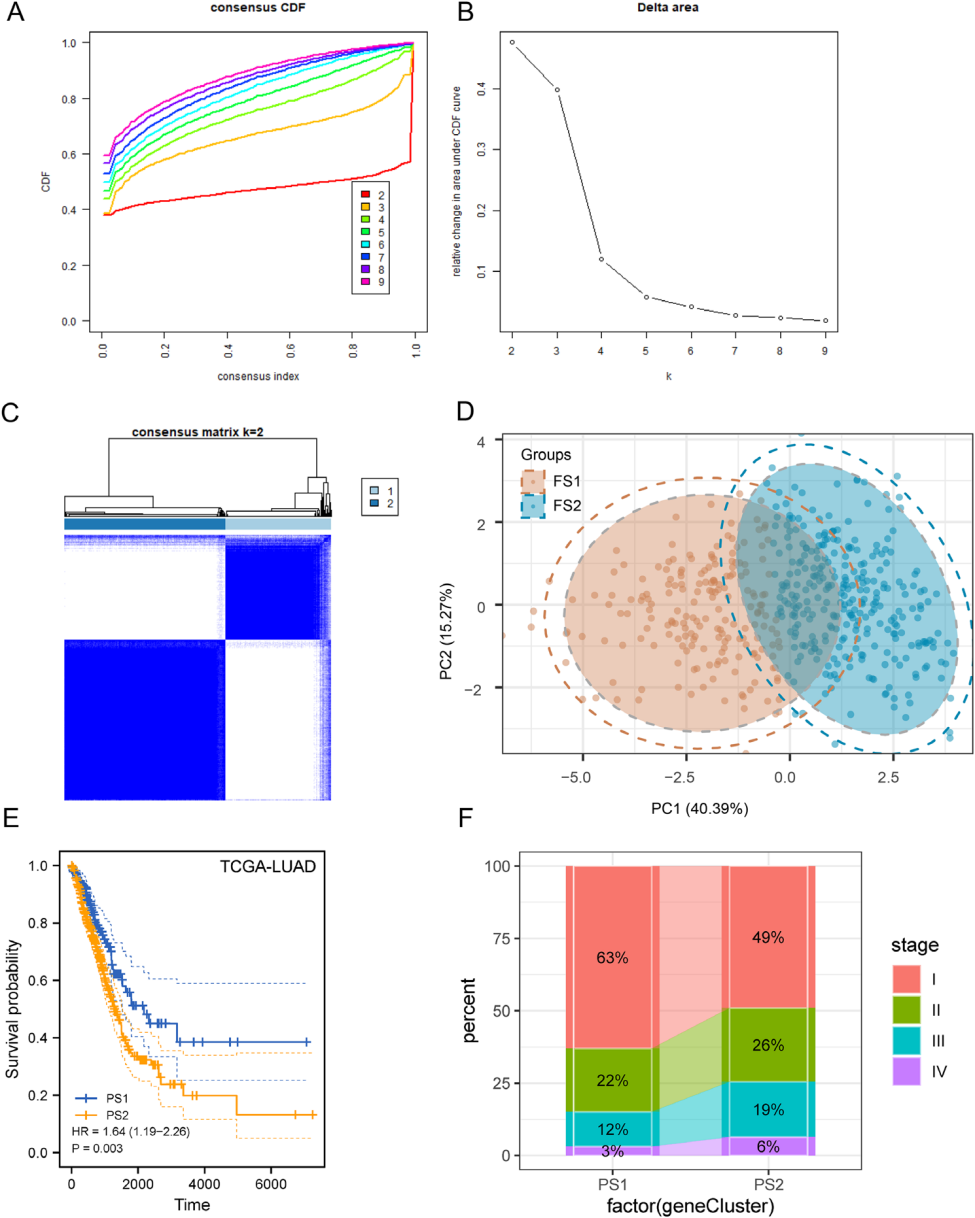
Discrimination of potential pyroptosis subtypes (PSs) of lung adenocarcinoma (LUAD). (**A**) The cumulative distribution function curve and (**B**) δ area of pyroptosis-related genes in The Cancer Genome Atlas (TCGA). (**C**) Sample clustering heat map. (**D**) A two-dimensional principal component analysis plot of the sample distribution. (**E**) Overall survival of patients with LUAD PSs in the TCGA group. (**F**) PS1 and PS2 in LUAD staging distribution. CDF, cumulative distribution function; LUAD, lung adenocarcinoma; OS, overall survival; PRG, pyroptosis-related gene; PS, pyroptosis subtype; TCGA, The Cancer Genome Atlas

Pyroptosis has been shown in recent years to be essential for tumor death and closely linked to tumor immunity. Pyroptosis isoforms could be applied to reflect the pyroptosis status in tumors and the tumor microenvironment; furthermore, they could help to clarify the optimal vaccination strategy for a patient. In this context, the expression profiles of 403 PRGs in TCGA-LUAD were investigated to establish proper consensus clusters. Based on functional triangle areas, k = 2 was selected. PRGs were stably clustered (Fig. 5A and C), and two PSs identified as PS1 and PS2 were obtained (Fig. 5D). PS1 was associated with a better prognosis, whereas PS2 had a lower probability of survival (Fig. 5E). The distribution of subtypes by tumor stage and grade revealed irregular clustering of patients with a diagnosis of the stage, with a higher proportion of patients with PS2 at intermediate-to-advanced stages (Fig. 5F). Consistent with the results of TCGA-LUAD group, GSE11969 could be divided into PS1 and PS2 (Supplementary Fig. 3A–3 C) and has prognostic relevance in this group (Supplementary Fig. 3D), with significant changes at different TNM stages (Supplementary Fig. 3E). In conclusion, pyroptosis typing can be applied to predict the prognosis of LUAD cases and was validated in different cohorts.

### Relationship between PSs and mutation status

**Fig. 6:**
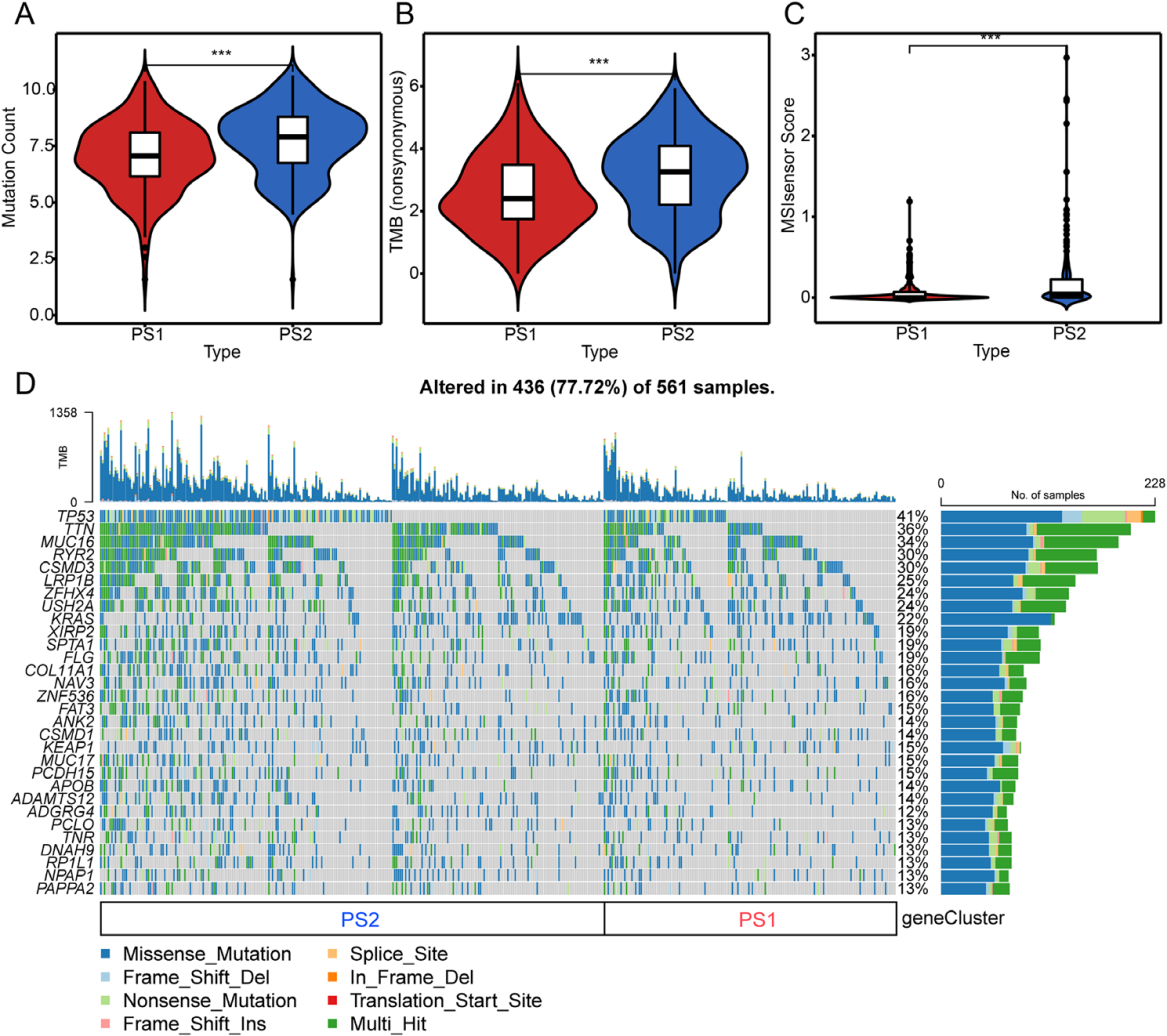
Relationship of pyroptosis subtypes with tumor mutation burden (TMB) and mutations. (**A**)–(**C**) Comparison of mutation count (**A**), TMB (**B**), and microsatellite instability (**C**) in different pyroptosis subtypes (PSs) of lung adenocarcinoma (LUAD). (**D**) LUAD waterfall plot showing mutation signature genes in different PSs (PS1 and PS2). Each grid represents a sample from the PS1 and PS2 subtypes. TMB stands for Tumor Mutational Burden, while the % and sample count reflect the mutation percentage and sample quantity of each gene in the PS1 and PS2 subtypes. ***P < 0.001. LUAD, lung adenocarcinoma; PS, pyroptosis subtype

A higher TMB is associated with better anti-cancer immunity. Therefore, this study determined the TMB, microsatellite instability (MSI), and the total number of mutations for each LUAD case based on the TCGA-LUAD mutation dataset and compared them for all PSs. As shown in Fig. 6A and C, the TMB and total mutation numbers were significantly lower in PS1 cases than in PS2 cases, whereas MSI was higher in PS2 cases. In addition, mutations were found in 436 of the 536 patient samples, with a mutation frequency of 77.72%. 30 genes, including TP53, TTN, and MUC16, showed different mutation statuses in different subtypes. The mutation frequency of TP53 was the highest in 41%. This was followed by TTN (36%), MUC16 (34%), RYR2(30%), and CSMD3 (30%) (Fig. 6D). These results suggested that TMB, MSI, and mutation number may serve as potential indicators for mRNA vaccines and that different mutation profiles exist in different PSs.

### Relationship between LUAD PS and immunomodulators

Previous studies have shown that ICP (e.g., PD-L1 and TIM-3) and ICD regulators (e.g., CALR) play important roles in regulating host anti-tumor immunity, thereby influencing the efficacy of mRNA vaccines, highlighting the link between pyroptosis and immune regulation. Hence, this study evaluated the differential expression of ICP regulators in the two PS groups. Among the 25 ICD genes identified in the TCGA-LUAD group, 12 had significantly different expression (Supplementary Fig. 4A). Furthermore, among the 21 ICD genes identified in the GSE11969 group, 15 had significantly different expression. In addition, a similar expression trend of differentially expressed ICD genes was observed in different subtypes: they were highly expressed in PS2 cases than in PS1 cases (Supplementary Fig. 4B). A total of 46 ICP genes were detected in the TCGA-LUAD cohort, with significantly different expression for 26 genes (Supplementary Fig. 4C). In comparison, 33 ICP genes were detected in the GSE11969 cohort, with significantly different expression for 21 genes (Supplementary Fig. 4D). Higher differential ICP gene expression was observed in the TCGA-LUAD group, which may be related to the larger sample size of this cohort. Supplementary Fig. 4A–4D show that the ICD and ICP gene expression trends were similar in both cohorts. However, different trends were observed in the different PS groups. In summary, pyroptosis typing may reflect ICD regulator and ICP levels, which can be used as a biomarker for mRNA vaccines. mRNA vaccines may be less effective in patients with high ICP and low ICD regulator levels.

### Cellular features of the pyroptosis subtype

**Fig. 7:**
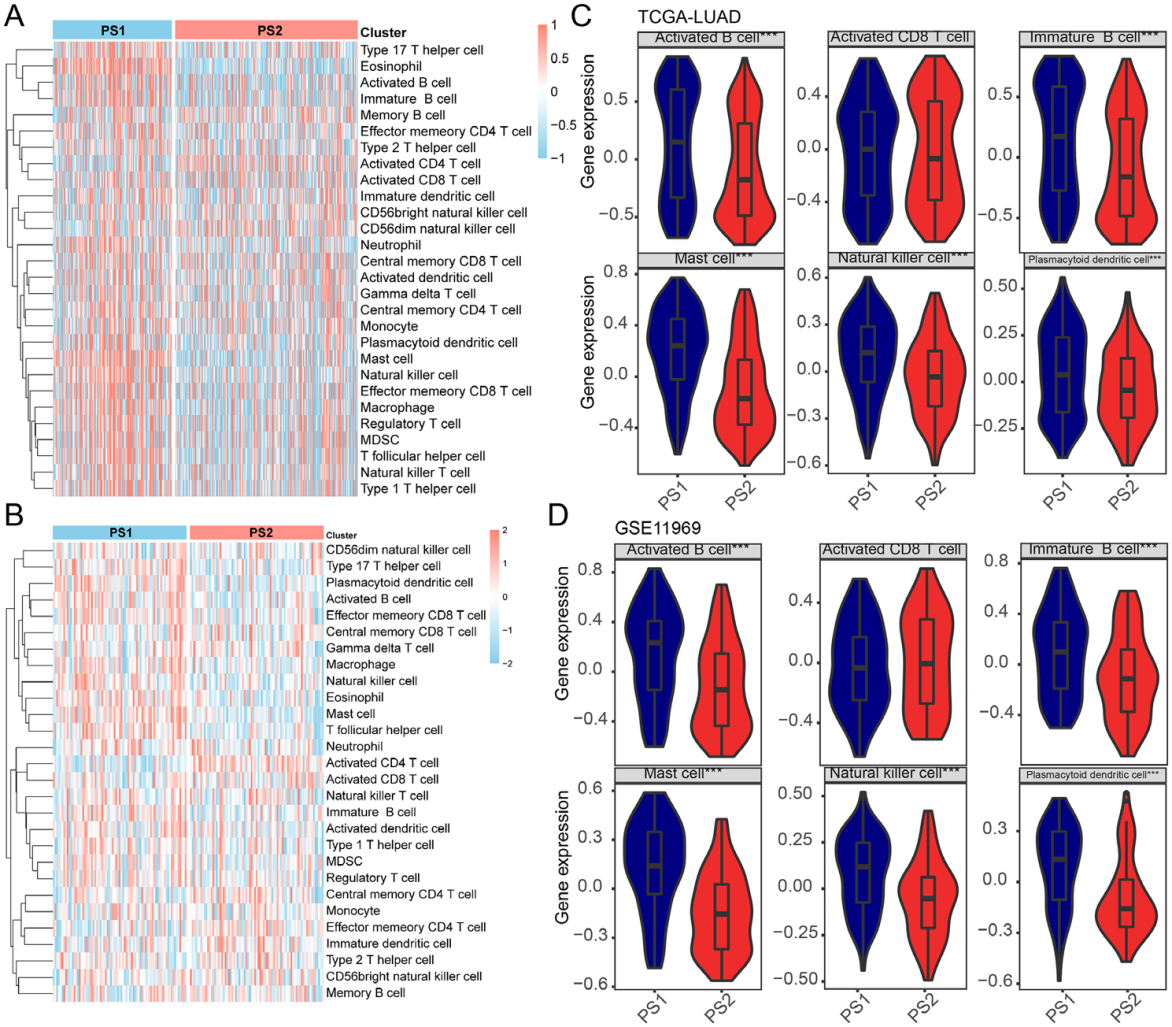
Cellular and molecular characterization of pyroptosis subtypes. (**A**) A heat map of the enrichment scores of 28 markers for lung adenocarcinoma (LUAD) immune subtypes in The Cancer Genome Atlas (TCGA)-LUAD and (**B**) the GSE11969 cohorts. (**C**) Scores of activated B cells, mast cells, activated CD8 + T cells, NK cells, and immature B cells in TCGA-LUAD cohorts. (**D**) Scores of activated B cells, mast cells, activated CD8 + T cells, NK cells, and immature B cells in the pyroptosis subtype 1 (PS1) and PS2 GSE11969 cohorts. *P < 0.05, **P < 0.01. LUAD, lung adenocarcinoma; PS, pyroptosis subtype

The response to mRNA vaccines is closely associated with the tumor’s immune status. Hence, the immune cell composition in the two PSs was determined by scoring 28 previously reported signature genes in the TCGA-LUAD and GSE11969 cohorts. Figure 7A and B show that immune cell components were classified into two clusters with significant differences in immune cell composition across subtypes. In addition, all six cell types scored, except for activated CD8 + T cells, showed higher scores in PS1 cases than in PS2 cases (Fig. 7C). The GSE11969 cohort showed a similar trend (Fig. 7D). K-M curves showed that six of the 22 immune cell types were associated with survival differences, including mast cells, activated CD8 + T cells, and immature B cells, all of which showed high scores for a good prognosis (Supplementary Fig. 5A–5 F). After calculating the immune score based on the ESTIMATE algorithm, it was found that more immune cell infiltration was present in patients with PS1 tumors compared with that in those with PS2 tumors (Supplementary Fig. 6A and 6B). Thus, PS1 is the immune “hot” phenotype, while PS2 is the immune “cold” phenotype. These results highlight that PS reflects the immune status of LUAD and could be used to identify patients suitable for mRNA inoculation. An mRNA vaccine containing these antigens could lead to relevant immune infiltration in immune “cold” PS2 tumors.

### Pyroptosis landscape of LUAD tumors

**Fig. 8:**
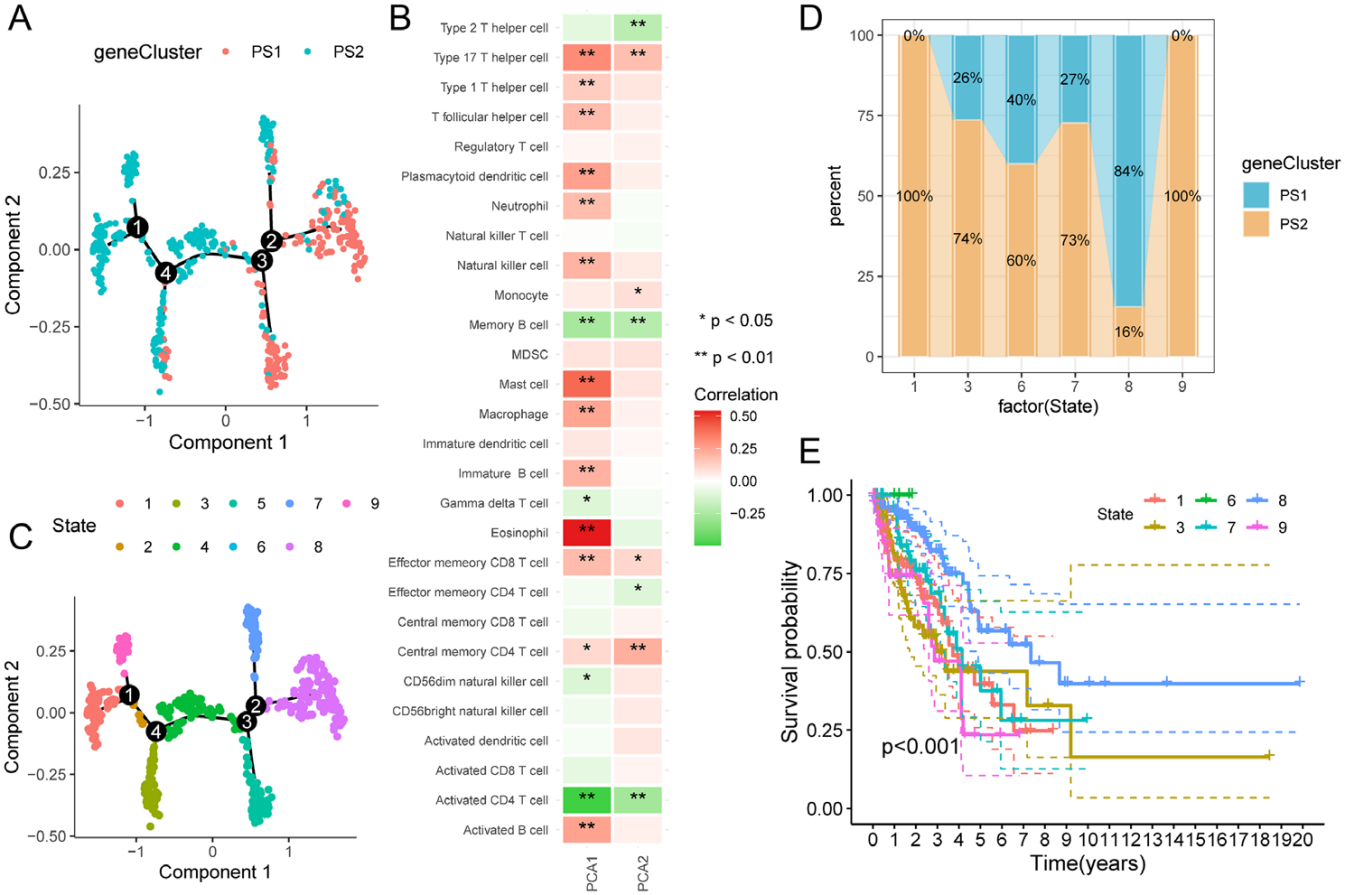
Pyroptosis landscape of lung adenocarcinoma (LUAD). (**A**) Pyroptosis landscape of LUAD. The position of each patient in the immune landscape, colored to correspond to the pyroptosis subtype identified above, refers to the overall features of the pyroptosis-related microenvironment. (**B**) Association of principal component analysis 1 / 2 with the immune module. (**C**) Reclassification of LUAD cases into states one, two, three, four, five, six, seven, eight, and nine according to their position. (**D**) The proportion of pyroptosis typing in patients with different states. (**E**) Prognostic curves of patients with different states. Differences can be seen between the prognosis of patients in different states. LUAD, lung adenocarcinoma; PCA, principal component analysis; PRME, pyroptosis-related microenvironment; PS, pyroptosis subtype

The pyroptosis profile of LUAD was established based on the pyroptosis gene-level profile of each patient (Fig. 8A). Figure 8B shows that the horizontal axis (PCA1) correlates with different immune cells, with the highest positive correlation being for activated B cells, eosinophils, macrophages, mast cells, and follicular T cells. The vertical axis (PCA2) is similar to the horizontal axis, with an inverse association with activated CD4 T cells, memory B cells and type 2 helper T cells and a positive association with central type memory T cells, effect memory CD8T cells, monocytes, and type 17 helper T cells. Within the same subtype, different intracluster heterogeneities were observed. Based on the trajectory position of the sample cluster, the entire sample was divided into nine states (Fig. 8C). Based on these states and positions, the states at the endpoints, including states one, three, six, seven, eight, and nine, were selected. The proportions in the different PSs are shown in Fig. 8D. Prognostic analysis showed significant differences in the survival curves of the six states, with state eight having the best prognosis (Fig. 8E). Altogether, the pyroptosis profile based on PSs could not accurately identify the pyroptosis status of each patient with LUAD and predict their prognosis. Further confirmation with more samples and typing validation may be required.

### Determination of the central gene for pyroptosis and the co-expression module of the pyroptosis gene in LUAD

**Fig. 9:**
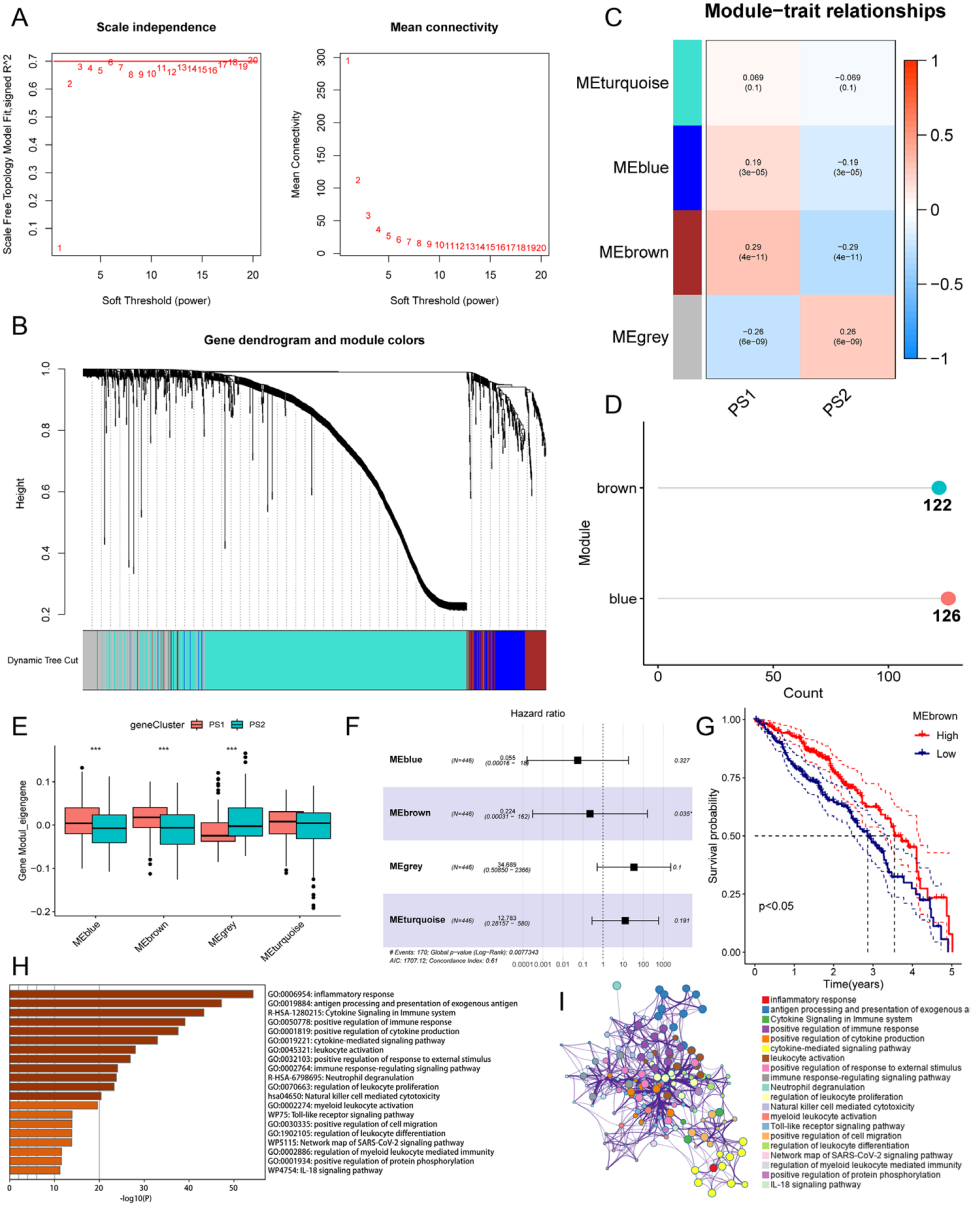
Discrimination of lung adenocarcinoma core pyroptosis genes. (**A**) Determination of optimal soft threshold values for WGCNA. (**B**) WGCNA clustering tree. (**C**) Four modules obtained through WGCNA. (**D**) Number of genes in modules with statistically significant differences. (**E**) Comparison of scores of pyroptosis subtypes in different modules. (**F**) Multifactorial Cox analysis of scores in different modules. (**G**) Prognostic survival curves of MEbrown genes, which are significantly different in high- and low-expression group. (**H**)–(**I**) Enrichment analysis of genes in the MEbrown module. PS, pyroptosis subtype

Identifying key PRGs could help oncologists determine whether patients are suitable for mRNA vaccines. To determine the key genes, the WGCNA of PRGs was established. For the scale-free network, the relevant soft threshold was set to six (Fig. 9A). The gene matrix was transformed into a topological matrix of adjacency matrices. At least 20 genes were selected for each module. The characteristic genes of each module were determined, and similar modules were merged. A total of four modules were finally obtained, where the gray module represents unassigned genes (Fig. 9B and C), and the numbers of genes in the blue and brown modules were 126 and 122, respectively (Fig. 9D). On comparing the Eigengene scores of the modules, it could be concluded that the PS1 scores were higher than the PS2 scores, except for the gray module (Fig. 9E and F). Subsequent prognostic analysis revealed a significant difference in scores and prognosis only for the brown module (Fig. 9G). Genes in the brown module were significantly enriched in immune-associated functions and pathways, including the inflammatory response, antigen processing and presentation of exogenous antigens, cytokine signaling, and positive regulation of cytokine production (Fig. 9H and I). Genes in the brown module included *FCGR2B, CD1C, LYZ, CGRT, TNFSF12, CD1A, FABP4, APOBEC3C, GRN, C3, TRIM22, CCL8, PLXNC1, RETN, FABP3, IL1RN, TNFSF13, TLR2, HAMP, CD74, CD1B*, and *ICAM2*. Therefore, key genes could be used as indicators to predict the prognosis of LUAD and identify patients suitable for mRNA vaccines.

### Key PRGs expression results in cell lines

The expression levels of 10 key PRGs in lung adenocarcinoma cell lines were verified by RT-qPCR from relative expression levels (Supplementary Fig. 7). The results showed that the expression levels of CSMD3, LRP1B, MUC16 and TTN in both lung adenocarcinoma cell lines were significantly increased (*p* < 0.05), CARD8, TP53 and ZFHX4 were significantly reduced in the expression levels of the two lung adenocarcinoma cell lines (*p* < 0.05), NLRP1 was only expressed in A549 cell lines (*p* < 0.05), NLRP3 expression trend was inconsistent in the two cell lines (*p* < 0.01), and NAIP expression levels in lung adenocarcinoma cell lines were not significantly different from normal human bronchial epithelial cells (*p* > 0.05).

## Discussion

LUAD is a typical non-small cell LC characterized by a high mortality rate. Continuous advances in different treatment methods have enhanced the prognosis of patients with LUAD. However, the OS of patients with LUAD remains poor [5, 6]. Therefore, effective treatments are required to improve the prognosis of these patients. As the molecular mechanism underlying LUAD pathogenesis is unclear, it is essential to investigate potential molecular mechanisms of action and identify new molecular diagnostic and prognostic biomarkers for LUAD. The wide application of expression profiling microarrays and second-generation sequencing technologies has increased the amount of high-throughput data available, and meaningful biomolecular markers could be identified through an in-depth analysis [32]. However, the molecular mechanisms underlying LUAD have not yet been fully elucidated.

In recent years, cancer vaccines have received increasing attention from researchers. mRNA vaccine therapy can induce an immune response in the body and plays a role in eliminating tumor cells through the tumor microenvironment, thus prolonging the survival of patients [33]. Many studies have shown cancer vaccine efficacy and therapeutic potential for tumor treatment [34–36]. However, they have not been widely used in clinical practice [37]. Furthermore, there have been few reports on the screening and study of tumor antigens associated with LUAD, which limits the promotion of mRNA vaccines for LUAD.

Inflammatory vesicles are multiprotein complexes that activate cellular inflammatory cytokines and induce pyroptosis [38]. CARD8, a sensor of inflammatory vesicles, has been reported to trigger leukemic cell pyroptosis by inhibiting dipeptidyl-peptidases, an inhibitory pathway that acts only on resting T lymphocytes (not activated T lymphocytes) [39, 40]. High NLRP1 expression has been shown to inhibit colon cancer cell growth [41]. NLRP1 and CARD8 are recognition receptors for forming inflammatory vesicles and produce two noncovalent polypeptide chains via the post-translational protein hydrolysis pathway [42]. NLRP1 can function as a molecular decoy to protect innate immune receptors and monitor the metabolic state of cells [42]. Targeting of inflammatory vesicles has potential as an anti-tumor therapy [43]. NAIP, NLRP1, and NLRP3 belong to the NOD-like receptor gene family, which is associated with innate immunity and tumors [44]. NAIP-NLRC4 inflammatory vesicles are associated with the pathogenesis of colorectal cancer, melanoma, glioma, and breast cancer [45]. NLRP3 vesicle-mediated inflammatory responses and are related to non-small cell lung cancer progression [46], which corroborates the OS analysis results of this study.

We selected two lung adenocarcinoma cell lines to verify the expression of the hub gene, and found that CSMD3, LRP1B, MUC16, TTN were highly expressed in lung adenocarcinoma cells, which provided effective evidence to support that they play a vital role in the development of lung adenocarcinoma and may be a new therapeutic target, which deserves more attention in follow-up research. We observed that the expression levels of CARD8, TP53 and ZFHX4 in the two lung adenocarcinoma cell lines were significantly reduced, which may indicate that the pyroptosis phenotype has been inhibited to a certain extent, suggesting a poor prognosis, which needs to be further verified in more cell lines and clinical tissue samples. Combining survival analysis results and RT-PCR test data, we speculate that CARD8 may be an RPG with more potential for tumor vaccine development.

Patients were classified into two PSs, PS1 and PS2, based on the expression profiles of 403 PRGs in the TCGA-LUAD, with patients with PS1 having a better prognosis. Four pyroptosis-associated antigens were screened: CARD8, NAIP, NLRP1, and NLRP3. We performed an analysis of the expression of CARD8, NAIP, NLRP1, and NLRP3 genes, and observed a significant correlation between these genes and antigen-presenting cells (APCs). This suggests their potential involvement in the activity of antigen-presenting cells, thereby indicating that these antigens may be presented to T cells by APCs and recognized by them [47]. Since these genes are involved in apoptosis, which is a form of programmed cell death that can lead to cellular rupture and demise, we hypothesize that these genes may initiate the process of apoptosis in tumor cells, consequently leading to cell death [42, 48]. The survival analysis results verified that high levels of these genes predicted a better prognosis. Therefore, this study provides a basis for screening sensitive populations and identifying new therapeutic targets for mRNA vaccines in patients with LUAD.

The prognostic results of this typing were validated using the GEO dataset. Significant differences were observed between patients with different PSs regarding TMB, somatic mutation rate, and MSI. Immune cell infiltration analysis showed that the overall level of immune cell infiltration was higher in patients with PS1 than in those with PS2, suggesting that the PS could effectively reflect the immune status of patients with LUAD. mRNA vaccines may stimulate immune cell infiltration by activating the body’s immune system, thus achieving tumor control. Previous studies combined genomic data from bladder cancer and head and neck squamous cell carcinomas to classify pyroptosis into different subtypes. The results further confirmed prognosis and immunotherapy sensitivity differences between patients with different tumor subtypes [49, 50]. This finding corroborates the findings of the present study. Therefore, it is suggested that patients with “cold” PS2 LUAD may be more suitable for treatment with antigen-containing mRNA vaccines.

All patients were divided into nine states based on the pyroptosis landscape of LUAD, among which eight states had the best prognosis. However, due to the insufficient sample size, the pyroptosis landscape mapped in this study could not accurately identify the pyroptosis status of each patient with LUAD. Therefore, more samples need to be included for an in-depth analysis.

This study had some limitations. First, the construction and validation of pyroptosis typing were based on retrospective public data. Therefore, prospective studies are necessary to confirm the accuracy and usefulness of our findings. Second, using a single phenotypic gene set to screen for antigens and populations sensitive to mRNA vaccines had unavoidable shortcomings, and some potentially significant phenotypic genes were inevitably missed. Simultaneously, some environmental and genetic factors closely related to the occurrence of LUAD were inevitably overlooked. Third, the biological function of PRGs in lung adenocarcinoma has not been verified in in vivo models and clinical samples, and further genetic and experimental studies and experimental verification of larger sample sizes are required. Pyroptosis prognostic models should be tested in actual clinical cases. Finally, further in-depth functional experiments are needed to verify the validity of the screened antigens. In future studies, we will further validate the roles of the selected vaccine-associated genes in treatment through clinical trials and basic experimental verification.

## Conclusions

Four potential antigens associated with tumor pyroptosis (CARD8, NAIP, NLRP1, and NLRP3) were screened as candidate molecules for future mRNA vaccine development. In addition, patients with PS2 LUAD were found to be suitable for mRNA vaccine treatment. To our knowledge, this study was the first to screen for therapeutic targets and sensitive populations based on the pyroptosis phenotype of LUAD cells, providing an important reference for developing LUAD mRNA vaccines.

### Electronic supplementary material

Below is the link to the electronic supplementary material.

Supplementary Material 1

Supplementary Material 2

## Data Availability

The datasets generated and analyzed during the current study are available in the GEO repository, https://www.ncbi.nlm.nih.gov/geo/query/acc.cgi?acc=GSE11969.

## Abbreviations

ANOVA: Analysis of variance
APC: Antigen-presenting cell
DEG: Differentially expressed gene
ICD: Immunogenic cell death
ICP: Immune checkpoint
LC: Lung cancer
LUAD: Lung adenocarcinoma
OS: Overall survival
PRG: Pyroptosis-related gene
TNM: Tumor Node Metastasis
PS: Pyroptosis subtype
TCGA: The Cancer Genome Atlas
TMB: Tumor mutation burden
RT-qPCR: Real-time quantitative polymerase chain reaction
GAPDH: Glyceraldehyde-3-phosphate dehydrogenase
