# Supplementary material for: Screening of novel tumor-associated antigens for lung adenocarcinoma mRNA vaccine development based on pyroptosis phenotype genes
Source: BMC Cancer. 2024 Jan 2;24:28. doi: 10.1186/s12885-023-11757-7 (PMC10763439; doi:10.1186/s12885-023-11757-7)
Supplement: Supplementary file 2 — Supplementary Material 2 [file 12885_2023_11757_MOESM2_ESM.docx]

**Tables**

Table S2 Table of Immune check point genes

|  | Gene symbol |
| --- | --- |
| Immune check point genes | *ADORA2A,BTLA,BTNL2,CD160,CD200,CD200R1,CD244,CD27,CD274,CD276,CD28,CD40,CD40LG,CD44,CD48,CD70,CD80,CD86,CTLA4,HAVCR2,HHLA2,ICOS,ICOSLG,IDO1,IDO2,KIR3DL1,LAG3,LAIR1,LGALS9,NRP1,PDCD1,PDCD1LG2,TIGIT,TMIGD2,TNFRSF14,TNFRSF18,TNFRSF25,TNFRSF4,TNFRSF8,TNFRSF9,TNFSF14,TNFSF15,TNFSF18,TNFSF4,TNFSF9,VTCN1* |

Table S3 Table of Immune cell death genes

|  | Gene symbol |
| --- | --- |
| Immune cell death genes | *ANXA1,CALR,CXCL10,EIF2A,EIF2AK1,EIF2AK2,EIF2AK3,EIF2AK4,FPR1,HGF,HMGB1,IFNA1,IFNA2,IFNAR1,IFNAR2,IFNB1,IFNE,IFNW1,LRP1,MET,P2RX7,P2RY2,PANX1,TLR3,TLR4* |

Table S4 Table of primers and their sequences for PCR analysis

| Primer | Sequence (5'→3') |
| --- | --- |
| H-GAPDH-F | GGAGCGAGATCCCTCCAAAAT |
| H-GAPDH-R | GGCTGTTGTCATACTTCTCATGG |
| H-TP53-F | CAGCACATGACGGAGGTTGT |
| H-TP53-R | TCATCCAAATACTCCACACGC |
| H-TTN-F | AAGCGTTGTGGTACTGGAGG |
| H-TTNR | TGGAAATCACCTGGCCATCC |
| H-CSMD3-F | TGACCCACACTACCTCCACT |
| H-CSMD3-R | TCGTAACTTGCACTCGCTGT |
| H-ZFHX4-F | TGCCAGTGGTCAGCTAATGG |
| H-ZFHX4-R | ACATGCACACTTAGGGCCTC |
| H-MUC16-F | ATCCTCCCCAGCTGAGACAT |
| H-MUC16-R | TGGCTGATGCTTCTTCCTGG |
| H-LRP1B-F | AATGGCGATTGACTGGCTCA |
| H-LRP1B-R | TTGGCGACATTCCCGTAGTC |
| H-CARD8-F | TGAGGAAGAGCTGCCGAGA |
| H-CARD8-R | GTCAACCAACAGTTTCCGTGA |
| H-NAIP-F | TAAACCTCAGATGTGAATTTCTTCG |
| H-NAIP-R | AGAGTCCAGCCGTAGTTCTTC |
| H-NLRP1-F | CTCGCCAATAAAGCGCACTC |
| H-NLRP1-R | TACTGAGCCACCAGGTACGA |
| H-NLRP3-F | TTGCTGTTTGACCCCGATGA |
| H-NLRP3-R | CTGTGTCACAAGGCTCACCT |

**Figures**


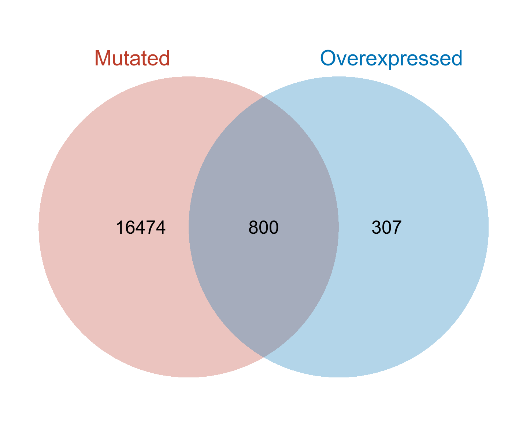


**Supplementary Figure 1**. Combining the results of mutated and overexpressed genes, 800 genes were identified as frequently mutated and upregulated cancer-related genes.


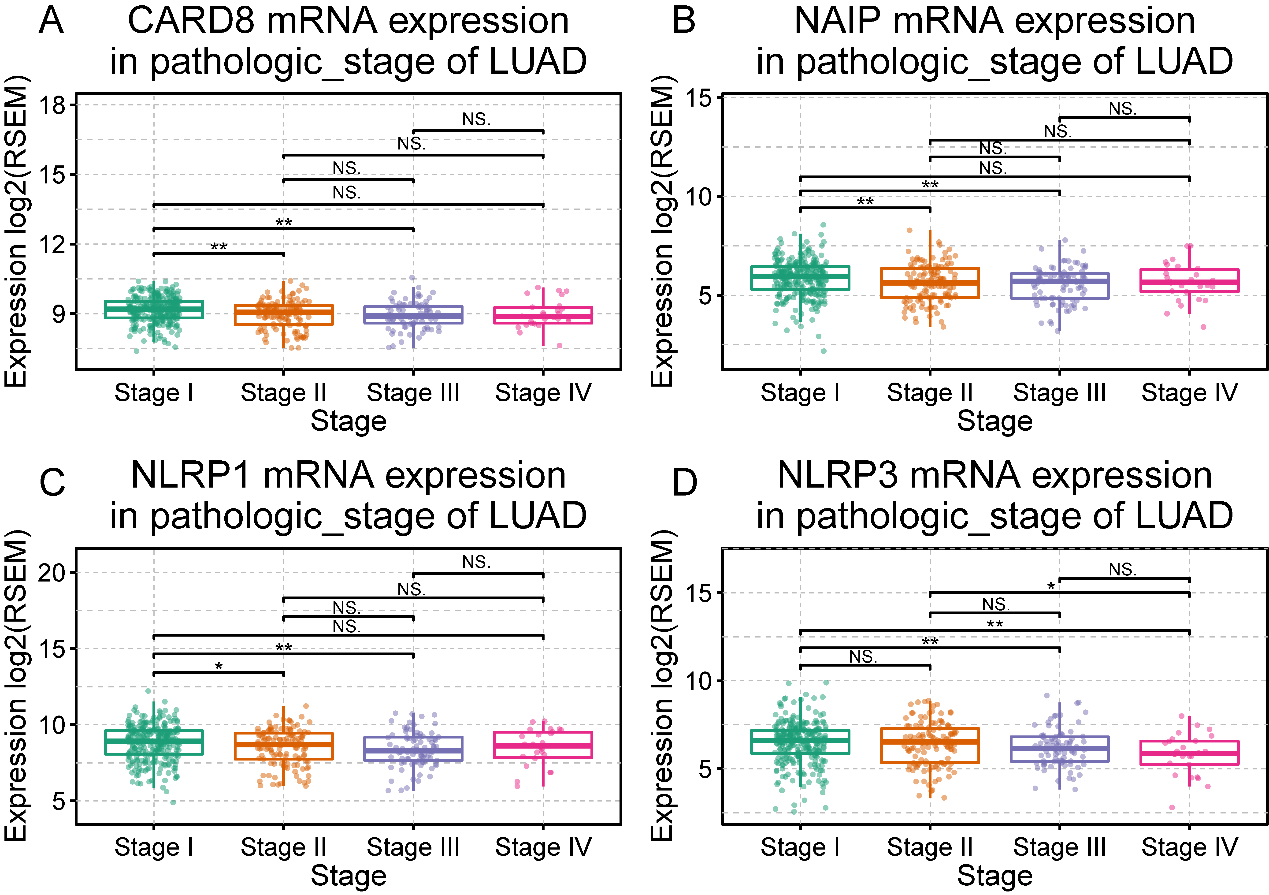


**Supplementary Figure 2. Relationship between potential tumor antigens and tumor Tumor Node Metastasis stage.** Expression of CARD8 (A), NAIP (B), NLRP1 (C), and NLRP3 (D) genes at different stages.


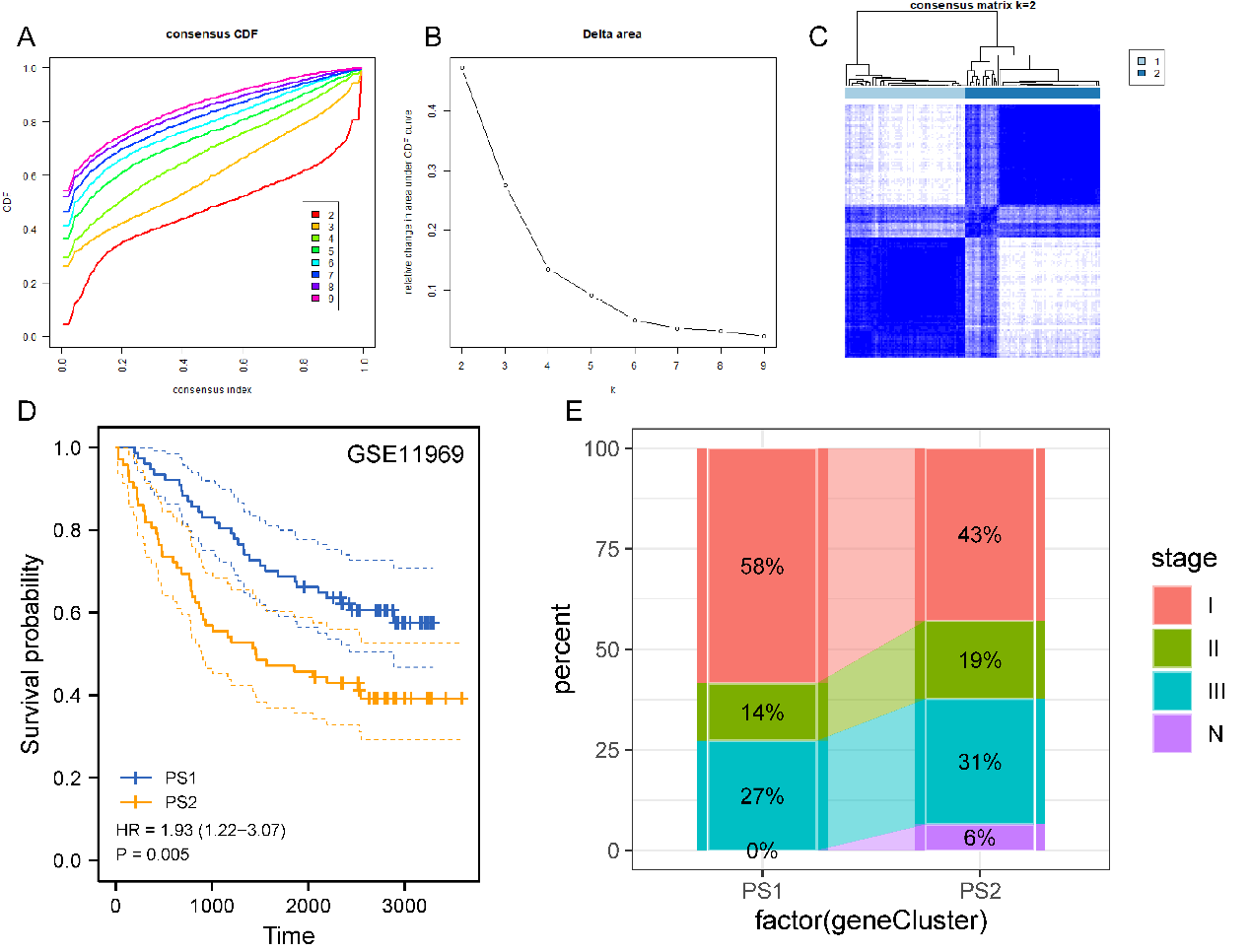


**Supplementary Figure 3. Identification of potential pyroptosis subtypes in lung adenocarcinoma (LUAD).** (A) Cumulative distribution function curve and (B) δ area of pyroptosis-related genes in GSE11969. (C) Two-dimensional principal component analysis plot of sample distribution at k = 2 in GSE11969. (D) Overall survival of patients with LUAD pyroptosis subtypes (PSs) in the GSE11969 cohort. (E) PS1 and PS2 in LUAD staging distribution. CDF, cumulative distribution function; LUAD, lung adenocarcinoma; OS, overall survival; PRGs, pyroptosis-related genes; PS, pyroptosis subtype.


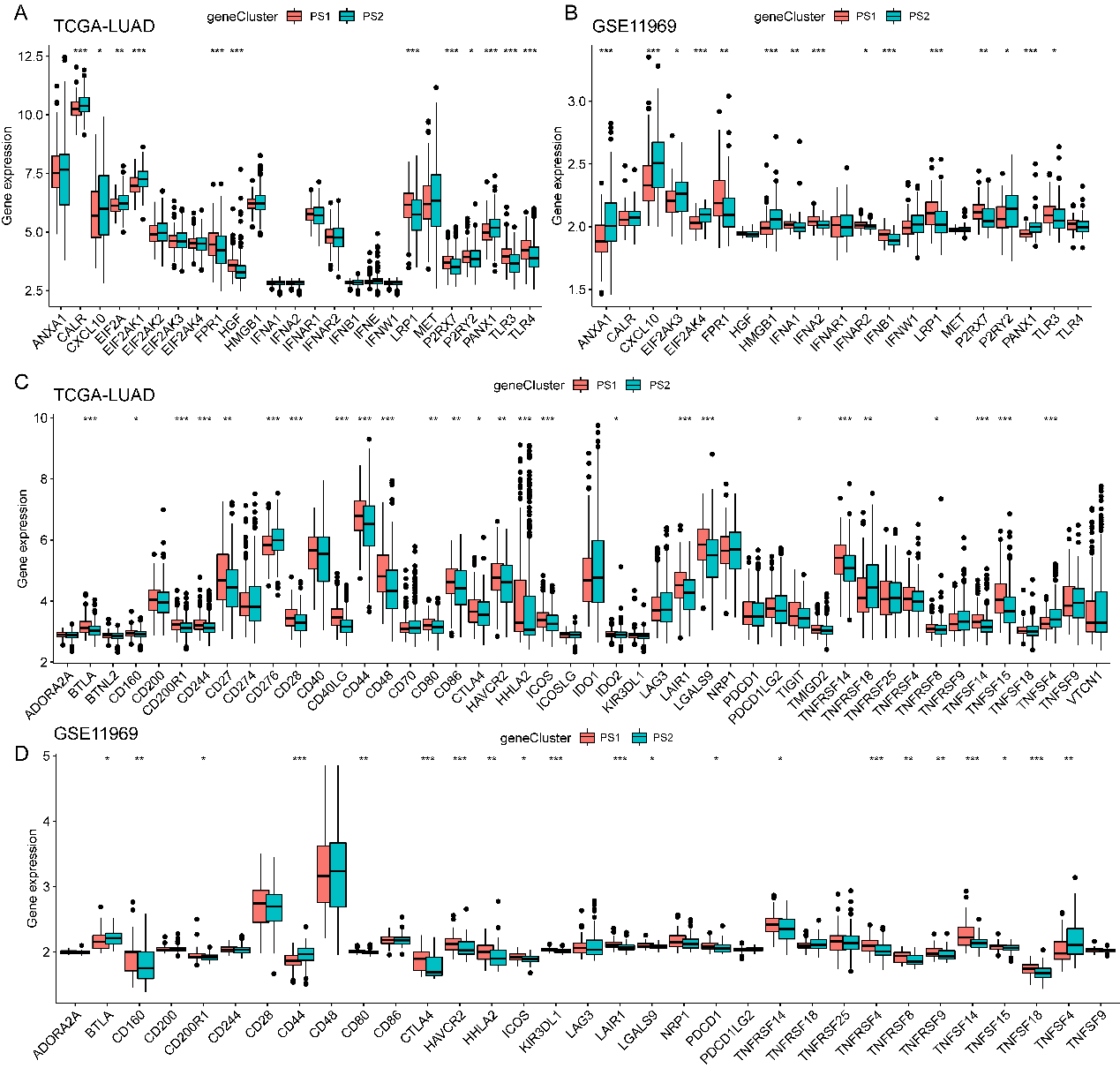


**Supplementary Figure 4. Association of pyroptosis subtypes with immune cell death (ICD) and immune checkpoint (ICP) regulatory genes.** (A)–(B) ICD gene expression difference between two pyroptosis subtypes (PSs) in The Cancer Genome Atlas (TCGA)-lung adenocarcinoma (LUAD) cohort (A) and the GSE11969 cohort (B). (C)–(D) Difference in ICP levels between two PSs in the TCGA-LUAD cohort (C) and the GSE11969 cohort (D). ICD, immune cell death; ICP, immune checkpoint; LUAD, lung adenocarcinoma; PS, pyroptosis subtype.


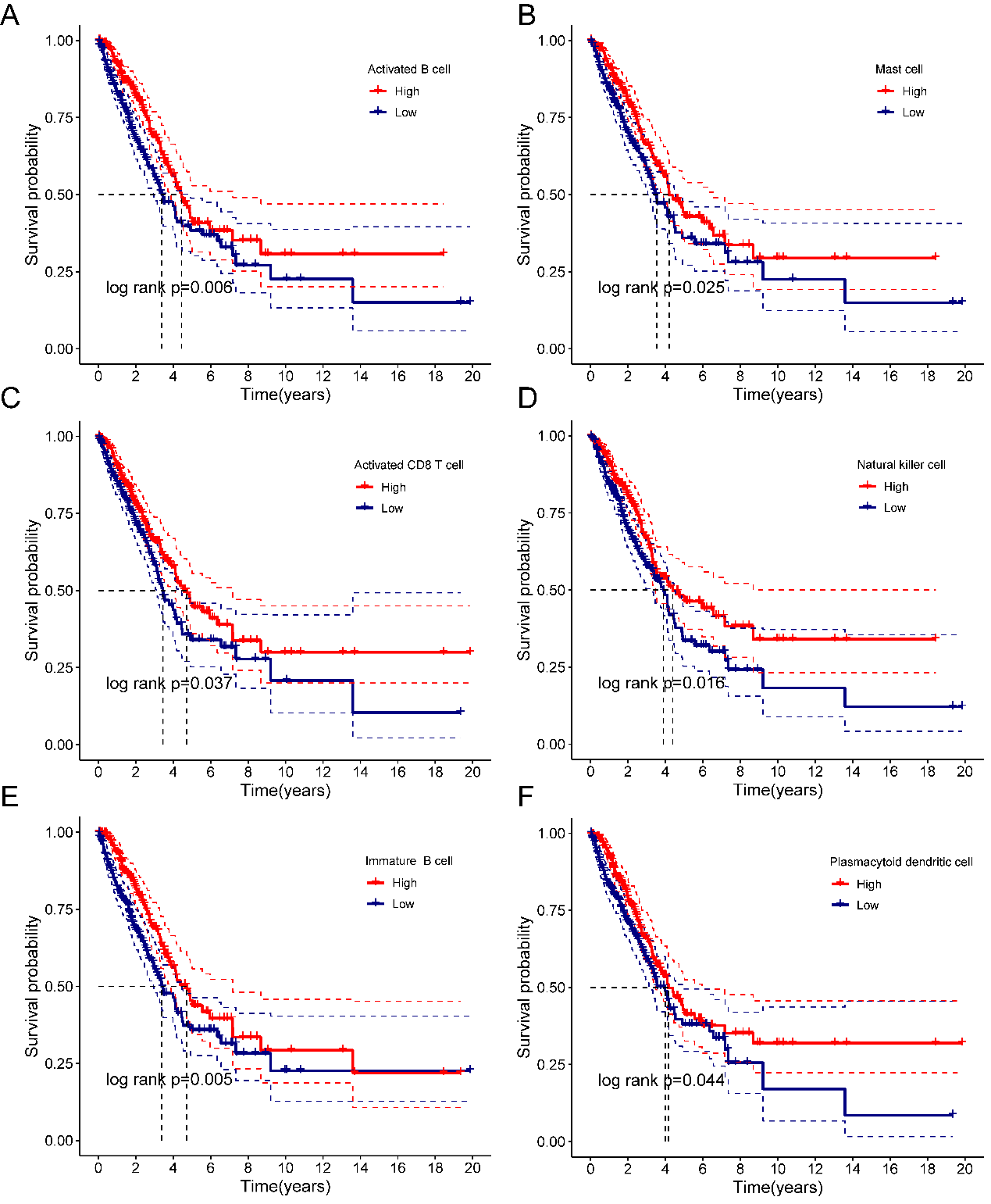


**Supplementary Figure 5. Immune cell survival differences.** (A)–(F) Different scores of activated B cells, mast cells, activated CD8+ T cells, NK cells, and immature B cells in The Cancer Genome Atlas-lung adenocarcinoma cohort have significant prognostic differences. LUAD, lung adenocarcinoma; TCGA, The Cancer Genome Atlas.


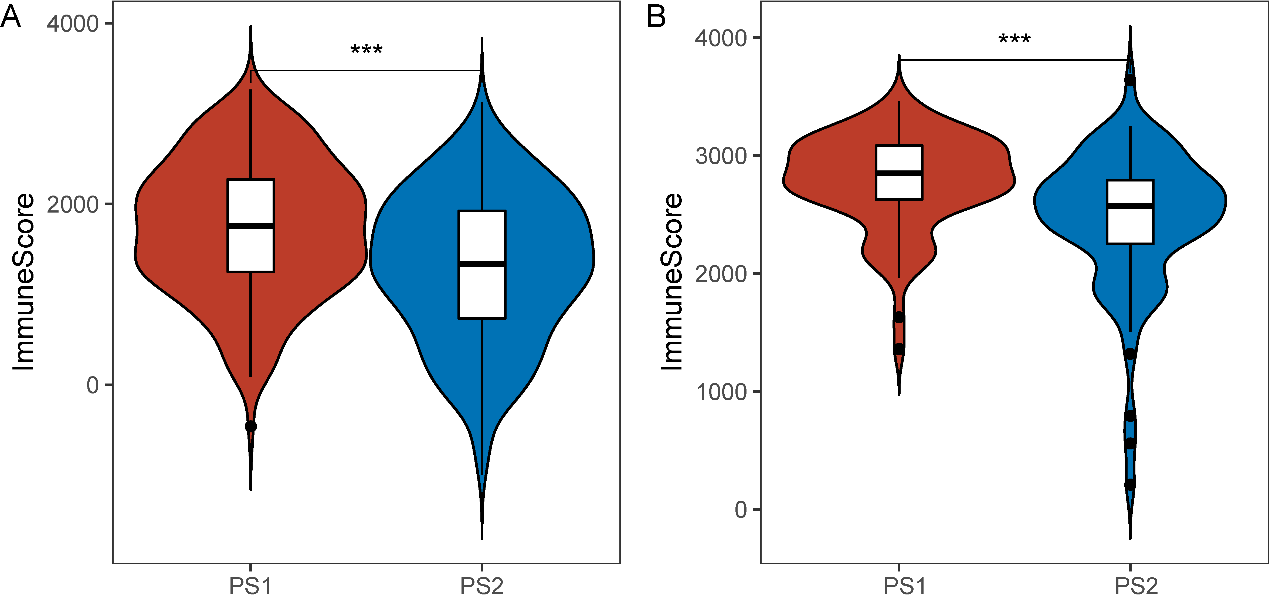


**Supplementary Figure 6. Comparison of immune scores calculated by ESTIMATE for different pyroptosis subtypes.** (A) Immune scores in The Cancer Genome Atlas-lung adenocarcinoma dataset. (B) Scores in the GSE11969 dataset. ***P < 0.001.


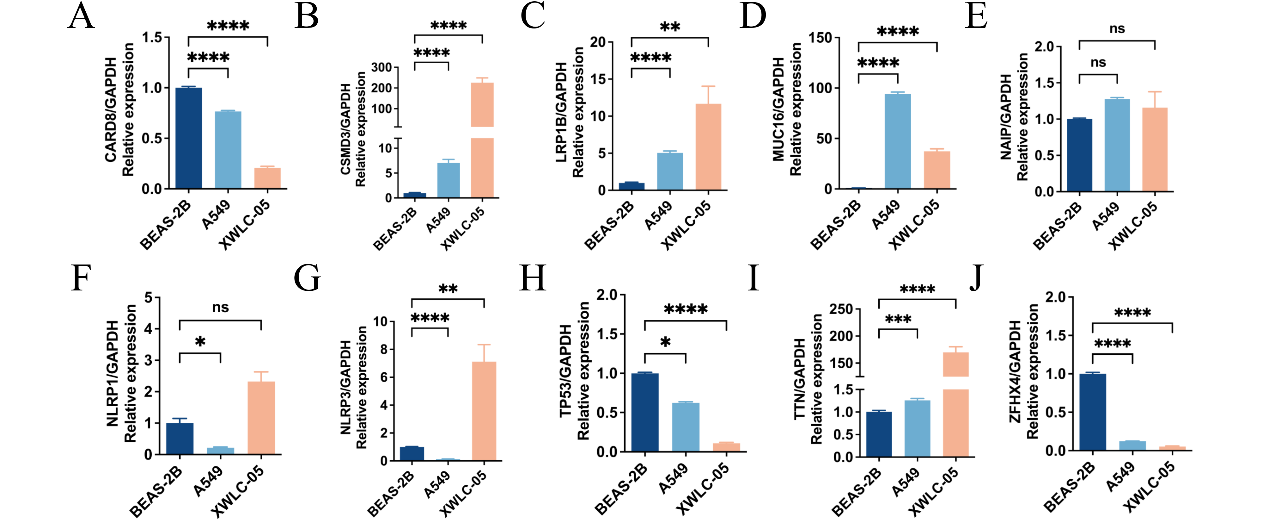


**Supplementary Figure 7. Histogram of the relative expression of key PRGs in cell lines.** (A-J) Histogram showing relative expression of 10 key PRGs in cell lines. Horizontal coordinates represent different cell lines, while vertical coordinates represent the relative expression of genes. BEAS-2B is normal human bronchial mucosal epithelial cell line, while A549 and XWLC-05 are human lung adenocarcinoma cell lines. *p < 0.05, **p < 0.005, ***p < 0.0005, and ****p < 0.0001. GAPDH, Glyceraldehyde-3-phosphate dehydrogenase.
